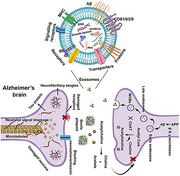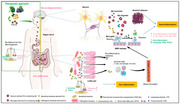# The role of microbiota‐derived exosomes in Alzheimer's disease and dementia progression and management

**DOI:** 10.1002/alz70855_102839

**Published:** 2025-12-23

**Authors:** Henry Demian Oyoyo, Jonas Ibekwe, Chukwuebuka Stanley Asogwa, Olarenwaju Timileyin, Ajibola Moshood, Fatola Ayomide, Olatunji Zion, Ogbeide Marvellous Aghahowa

**Affiliations:** ^1^ Mission Brain Ibadan, Ibadan, Oyo ‐ State, Nigeria; ^2^ University Of Ibadan, Ibadan, Oyo ‐ State, Nigeria; ^3^ College Research and Innovation Hub, Ibadan, Oyo ‐ State, Nigeria; ^4^ College of Medicine University of Ibadan, Ibadan, Oyo ‐ State, Nigeria; ^5^ College Research and Innovation Hub, Ibadan, Nigeria; ^6^ University Of Benin, Benin, Edo ‐ State, Nigeria; ^7^ College of Medicine University of Benin, Benin, Edo ‐ State, Nigeria

## Abstract

**Background:**

Alzheimer's disease (AD) and dementia are complex neurodegenerative disorders characterized by progressive cognitive decline. Recent research has highlighted the significance of the microbiota‐gut‐brain axis in modulating brain health, with microbiota‐derived exosomes emerging as key players in intercellular communication. These exosomes, small extracellular vesicles, can influence neuroinflammation, amyloid‐beta dynamics, and neuroprotection, potentially impacting Alzheimer's disease pathology. This narrative review aims to explore the current understanding of how microbiota‐derived exosomes influence the progression of Alzheimer's disease and dementia, and to assess their therapeutic potential in disease management. This study synthesizes evidence from recent studies to provide insights into the mechanisms by which these exosomes modulate neuroinflammatory responses and contribute to neurodegenerative processes.

**Method:**

A comprehensive literature search was conducted across major databases, including PubMed, Cochrane library, and Scopus to identify relevant studies on microbiota‐derived exosomes and their role in Alzheimer's disease. The search strategy included keywords related to microbiota, exosomes, Alzheimer's disease, dementia, gut‐brain‐axis and neurodegeneration. Studies were selected based on their relevance to the topic and the quality of evidence provided.

**Result:**

The review highlights that microbiota‐derived exosomes can cross the blood‐brain‐barrier and modulate microglial activation and inflammatory responses, potentially influencing Alzheimer's disease progression. Additionally, these exosomes may affect amyloid‐beta aggregation and clearance, contributing to disease pathology. Emerging evidence from previous studies suggest that microbiota‐derived exosomes could serve as potential biomarkers for early Alzheimer's disease detection and monitoring.

**Conclusion:**

This study underscores the importance of microbiota‐derived exosomes in understanding Alzheimer's disease and dementia progression. The findings suggest the gut‐brain axis, mediated in part by these exosomes, represents a promising avenue for future research and effective therapeutic interventions as targeting these exosomes could offer novel therapeutic strategies for managing neurodegenerative diseases. However, further studies are needed to fully elucidate the mechanisms involved and translate these findings into clinical applications. This work contributes to the growing body of evidence supporting the microbiota‐gut‐brain axis as a critical area of investigation for neurodegenerative disease research.